# Gastrointestinal investigation of parasites and Enterobacteriaceae in loggerhead sea turtles from Italian coasts

**DOI:** 10.1186/s12917-019-2113-4

**Published:** 2019-10-25

**Authors:** Antonino Pace, Laura Rinaldi, Davide Ianniello, Luca Borrelli, Giuseppe Cringoli, Alessandro Fioretti, Sandra Hochscheid, Ludovico Dipineto

**Affiliations:** 1 Department of Veterinary Medicine and Animal Productions, University Federico II, via Delpino 1, 80137 Naples, Italy; 2 Marine Turtle Research Centre, Stazione Zoologica Anton Dohrn, via Nuova Macello 16, 80055 Portici, Na Italy; 3 Task Force on Microbiome Studies, University Federico II, Naples, Italy

**Keywords:** *Caretta caretta*, Endoparasites, Enterobacteriaceae, FLOTAC, Mediterranean Sea

## Abstract

**Background:**

*Caretta caretta* is the most abundant sea turtle species in the Mediterranean, and studies on this species have vastly expanded during recent years, including those investigating gut bacterial and parasitic communities. Members of these communities have been reported with variable prevalence and pathogenicity, mainly depending on their host and environment (e.g. lifespan, distribution, habitat, diet, health status and stressors). Indeed, many species commonly inhabiting the sea turtle gastrointestinal tract exhibit an opportunistic behaviour.

This study aimed to provide baseline data on enterobacterial and parasitic composition, through bacteriological culture-based methods and the FLOTAC parasitological technique, in cloacal and faecal samples of 30 live *Caretta caretta*, examined upon their arrival at the Marine Turtle Research Centre (Portici, Italy).

**Results:**

Enterobacteriaceae were isolated in 18/23 cloacal samples (78.3%), with *Citrobacter* and *Morganella* as the most common genera, followed by *Proteus*, *Enterobacter*, *Providencia*, and *Hafnia*. Parasitic elements were detected in 11/30 faecal samples (36.7%), with *Enodiotrema*, *Rhytidodes*, and *Eimeria* as most common genera, followed by *Pachypsolus* and *Cymatocarpus*. Additionally, *Angiodyctium* is reported for the first time in this host. The majority (47.8%) of sea turtles hosted exclusively Enterobacteriaceae, whereas 30.4% hosted both parasites and Enterobacteriaceae; the remaining 21.8% hosted neither of the agents.

**Conclusions:**

Bacteria and parasites evaluated in the present study are common in Mediterranean loggerhead sea turtles, with slight differences between the western and eastern basin. Although naturally present in the gastrointestinal system of free-living sea turtles, their relationship with these hosts might range from mutualism to parasitism. Indeed, members of the gut community might express their pathogenic potential in immune-compromised animals, such as those in rehabilitation facilities. Therefore, it is advisable to include in the standard work-up of rescued sea turtles a screening procedure for such opportunistic agents, in order to better evaluate the animal’s health status and achieve timely intervention with appropriate treatment, thus improving rehabilitation. Furthermore, data collected from free-living sea turtles represent a starting point for investigating wild populations. However, further studies are needed to clarify the differences between sea turtle’s normal gut microbiome and pathobiome.

## Background

*Caretta caretta* is the most abundant sea turtle species in the Mediterranean Sea, using both the eastern and western basins for nutrition, reproduction and overwintering [10, 12]. Studies on this species started with conservation programs and have vastly expanded during the last decades [10]. In particular, the knowledge of the health status of wild populations, including what infectious agents are present, has received increasing attention [19]. Several studies have investigated bacterial and parasitic communities of sea turtles, especially in the gastrointestinal tract [7, 13, 29, 31, 32, 50–53, 64].

Many bacteria are regarded as common gut inhabitants in sea turtles, although they exhibit an opportunistic behaviour, becoming pathogenic in immune-compromised animals [3, 4, 26].

Indeed, Proteobacteria, which have been reported as the most abundant phylum in rescued sea turtles by recent molecular studies [3, 4, 41], might establish symbiotic or pathogenic relations with their hosts, although their precise role within the sea turtle gastrointestinal tract is still unknown [3]. Within this phylum, members of the family Enterobacteriaceae might play an important role as opportunistic agents, since they were recovered in cloacal samples of healthy and debilitated sea turtles alike, through both molecular and culture-dependent methods [3, 7, 26, 33, 51, 52].

As regards to parasites, both ecto and endo-parasites have been described in sea turtles, with relationship ranging from parasitic, to mutualistic or symbiotic [29, 61]. Sea turtles usually act as definitive hosts, but sometimes they serve as intermediate (e.g. Cestoda) or paratenic hosts (e.g. *Anisakis* spp.) [29, 54]. Different biological and ecological factors (i.e. lifespan, feeding habits, site fidelity and migration patterns) influence the composition and richness of endoparasitic communities [13, 36, 47, 61, 64]. In particular, loggerhead sea turtles are mainly susceptible to digenetic trematodes and nematodes [29, 61], with invertebrates and fish as intermediate hosts [17, 28], whereas only a few protozoa have been reported [29, 63]. In the Mediterranean, parasitological studies examined over 300 loggerhead sea turtles, evidencing a relative depauperate helminth community with strong dissimilarities among locations [5, 28, 29, 34, 40, 47, 57]. Sea turtle endoparasites may affect various organs, but the gastrointestinal helminths are the most commonly recovered [39, 40, 61]. In healthy hosts, endoparasites rarely cause problems, although some exceptions have been reported [27, 59]. Furthermore, stress associated with disparate causes (e.g. diseases, environmental imbalances, migration, nesting) could make sea turtles vulnerable to higher parasite intensities, and consequently manifest disease [13, 14, 24, 55, 59, 61, 67]. Diagnosis of helminth infections is usually achieved through egg detection, both using flotation and sedimentation, or through collection of adult endoparasites [13, 29, 35, 61]. Whatever the technique might be, almost all of the parasitological surveys in the literature made use of samples obtained from carcasses, because of the difficulty of accessing and sampling live sea turtles [13, 24, 29, 36, 49, 64].

The gastrointestinal tract, in sea turtles as well as in other organisms, might be rightly considered as an ecosystem where bacteria, viruses, protozoa, fungi and endoparasites co-exist [44, 45]. The interplay between host, parasites and microbiota has attracted much attention and several studies have been conducted on different species [6, 20, 42]. On the other hand, the composition of the sea turtle gut microbiome has drawn attention only recently [1, 3, 4, 41], requiring further investigations to better understand the pathogenic or symbiotic relationship between parasites, bacteria and these hosts. This study aimed to provide baseline data on enterobacterial and parasitic composition, through bacteriological culture-based methods and the FLOTAC parasitological technique, in cloacal and faecal samples of live loggerhead sea turtles from the Italian coasts, thus supporting the existing literature and improving the rehabilitation of this endangered species.

## Results

**Fig. 1:**
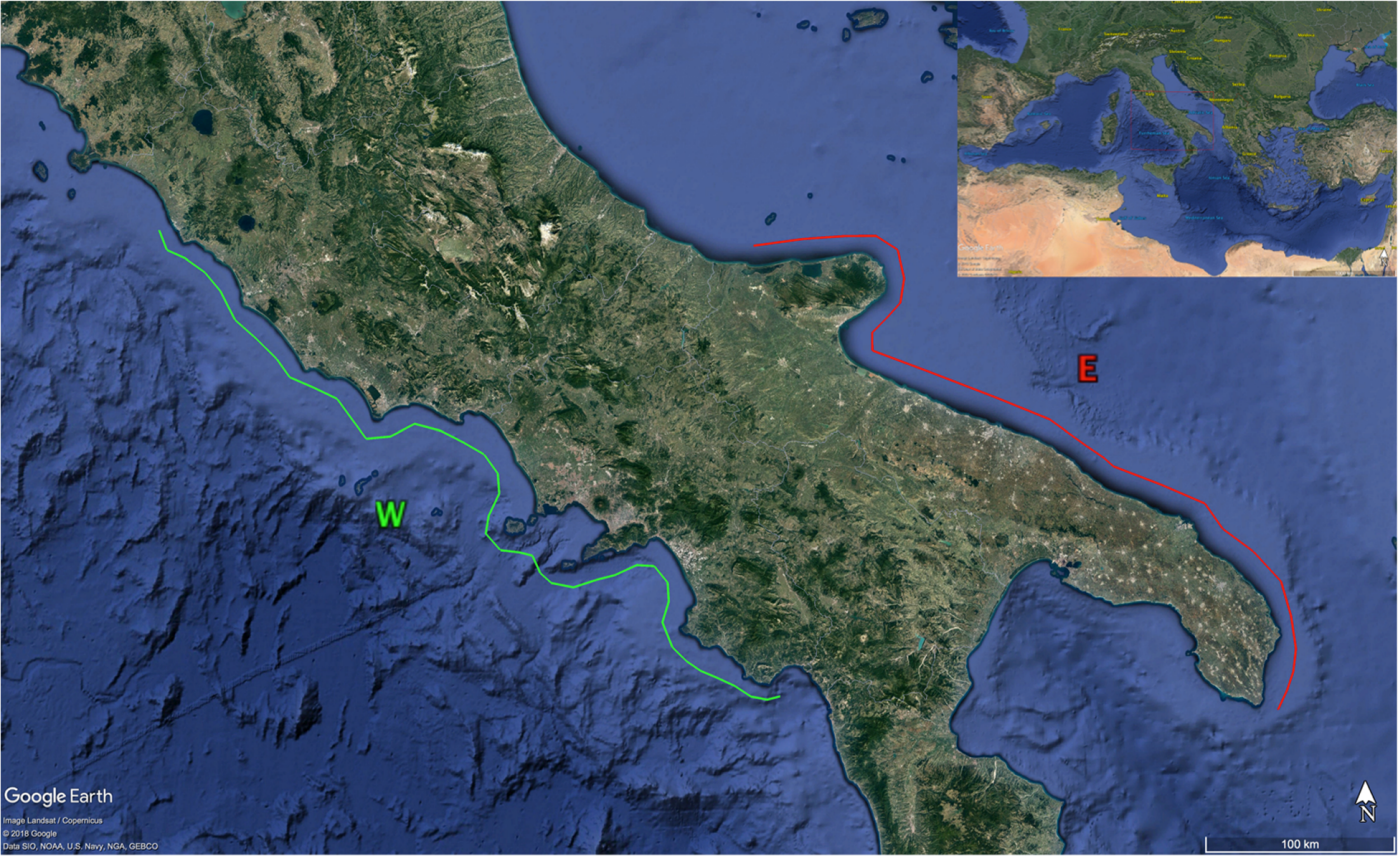
Area of sea turtle recovery. Twenty sea turtles were recovered along the southwestern coast of Italy (area W, green line) and ten along the southeastern coast of Italy (area E, red line). (Map data: Google Earth, Image Landsat/Copernicus© 2018 Google Data SIO, NOAA, U.S. Navy, NGA, GEBCO; Inset Map data: Google Earth Image Landsat/Copernicus©2018 Google US Dept of State Geographer© 2009 GeoBasis-DE/BKG. Images have been modified and assembled according to Google permission and attribution guidelines)

Cloacal and faecal samples were collected from 30 loggerhead sea turtles (25 juveniles and 5 adults), recovered along the southwestern (n. 20) and southeastern (n.10) coasts of Italy (Fig. 1). The bacteriological and parasitological results related to each sea turtle are summarized in Table 1.

**Table 1:** Parasites and Enterobacteriaceae detected in 30 *Caretta caretta* recovered along the western and eastern coasts of southern Italy

Turtle ID	Faecal samples		Cloacal swabs
	Parasites	EPG/OPG^a^	Bacteria^b^
WEST 01	Trematodes		*Citrobacter* spp.
	*Angiodyctium* spp.	8	
	Coccidia		
	*Eimeria carettae*	48	
WEST 02	Negative	–	Negative
WEST 03	Negative	–	*Proteus* spp.
WEST 04	Negative	–	*Citrobacter* spp.; *Morganella morganii*
WEST 05	Negative	–	*Citrobacter* spp.; *Morganella morganii*
WEST 06	Negative	–	*Citrobacter* spp.; *Morganella morganii*
WEST 07	Negative	–	*Citrobacter* spp*.*; *Morganella morganii*; *Proteus* spp.
WEST 08	Negative	–	*Enterobacter* spp.; *Escherichia coli*
WEST 09	Trematodes		*Citrobacter* spp.; *Proteus* spp.
	*Cymatocarpus* spp.	35	
WEST 10	Negative	–	Negative
WEST 11	Negative	–	*Citrobacter* spp.; *Morganella morganii*
WEST 12	Negative	–	*Citrobacter* spp.; *Morganella morganii*
WEST 13	Coccidia		*Citrobacter* spp.; *Enterobacter* spp.
	*Eimeria carettae*	50	
WEST 14	Negative	–	Negative
WEST 15	Trematodes		*Citrobacter* spp.; *Escherichia coli*
	*Enodiotrema* spp.	64	
WEST 16	Negative	–	*Citrobacter* spp.; *Enterobacter* spp.; *Morganella morganii*; *Proteus* spp.; *Providencia rettgeri*
WEST 17	Coccidia		c
	*Eimeria carettae*	70	
WEST 18	Trematodes		c
	*Angiodyctium* spp.	48	
WEST 19	Negative	–	c
WEST 20	Trematodes		c
	*Enodiotrema* spp.	14	
EAST 01	Negative	–	Negative
EAST 02	Trematodes		*Hafnia alvei*; *Morganella morganii*; *Providencia rettgeri*
	*Rhytidodes* spp.	140	
EAST 03	Negative	–	*Citrobacter* spp.
EAST 04	Negative	–	Negative
EAST 05	Trematodes		*Citrobacter* spp.
	*Rhytidodes* spp.	24	
EAST 06	Negative	–	*Citrobacter* spp.; *Morganella morganii*
EAST 07	Trematodes		*Morganella morganii*
	*Pachypsolus* spp.	180	
EAST 08	Trematodes		c
	*Enodiotrema* spp.	80	
	*Rhytidodes* spp.	112	
EAST 09	Negative	–	c
EAST 10	Negative	–	c

^a^ EPG/OPG, eggs/oocysts per gram of faeces

^b^ Bacterial species were confirmed when the API identification matched at least ‘very good ID’ (%id> 99.0 and T > 0.5)

^c^ Sample excluded from analysis due to suspected contamination

The bacteriological survey revealed that 18 out of the 23 (78.3%) loggerhead sea turtles hosted members of the Enterobacteriaceae family. The remaining seven cloacal swabs, although tested, were excluded for technical reasons (see Methods). The majority of turtles hosted either one species (5/18, 27.8%) or two (10/18, 55.6%); few animals hosted three (2/18, 11.1%) or more species (1/18, 5.6%), as reported in Table 1. The species most frequently recovered and their prevalence is reported in Table 2. *Enterobacter* spp., *E. coli* and *Proteus* spp. were detected only in samples from sea turtles recovered along the southwestern coast of Italy, whereas *Hafnia alvei* was detected only in samples from sea turtles recovered along the southeastern coast (Table 2).

**Table 2:** Prevalence of Enterobacteriaceae species isolated from 23 cloacal swabs of *Caretta caretta*

Bacterial species	Positive animals (n.)	Prevalence (95% CI)^a^		
		Total (n. 23)	West (n. 16)	East (n. 7)
*Citrobacter* spp.	14	60.9% (40.8–77.8%)	68.8% (44.4–85.8%)	42.9% (15.8–75%)
*Morganella morganii*	10	43.5% (25.6–63.2%)	43.8% (23.1–66.8%)	42.9% (15.8–75%)
*Proteus* spp.	4	17.4% (7.0–37.1%)	25% (10.2–49.5%)	0% (0–35.4%)
*Enterobacter* spp.	3	13.0% (4.5–32.1%)	18.8% (6.6–43%)	0% (0–35.4%)
*Escherichia coli*	2	8.7% (2.4–26.8%)	12.5% (3.5–36%)	0% (0–35.4%)
*Providencia rettgeri*	2	8.7% (2.4–26.8%)	6.3% (1.1–28.3%)	14.3% (2.6–51-3%)
*Hafnia alvei*	1	4.4% (0.8–21.0%)	0% (0–19.4%)	14.3% (2.6–51-3%)

^a^ 95% CI, 95% Confidence interval

The parasitological survey revealed that 11 out of the 30 (36.7%) loggerhead sea turtles hosted parasites, with different parasitic burden, measured in eggs/oocysts per gram of faeces (EPG/OPG) (Table 1). Exclusively trematode eggs and protozoa oocysts were detected: their prevalence and mean parasitic burden are reported in Table 3. In most of the turtles (9/11), just one parasite species was detected from each animal; only in two cases there was a co-infection of parasites (Table 1). *Eimeria* oocysts and *Cymatocarpus* eggs were detected only in faeces of sea turtles recovered along the southwestern coast of Italy, whereas *Pachypsolus* and *Rhytidodes* eggs were detected only in faeces of sea turtles recovered along the southeastern coast (Table 1).

**Table 3:** Prevalence and mean parasitic burden of parasites detected from 30 faecal samples of *Caretta caretta*

Parasite species	Positive animals (n.)	Prevalence (95% CI)^a^	Mean EPG/OPG^b^ (±SE)^c^
Coccidia			
*Eimeria carettae*	3	10% (3.5–25.6%)	56 (±7.0)
Trematodes			
*Enodiotrema* spp.	3	10% (3.5–25.6%)	52.7 (±19.9)
*Rhytidodes* spp.	3	10% (3.5–25.6%)	92 (±34.9)
*Angiodyctium* spp.	2	6.7% (1.9–21.3%)	28 (±20.0)
*Cymatocarpus* spp.	1	3.3% (0.6–16.7%)	35
*Pachypsolus* spp.	1	3.3% (0.6–16.7%)	180

^a^ 95% CI, 95% Confidence interval

^b^ EPG/OPG, eggs/oocysts per gram of faeces

^c^ Standard Error

Concerning the correlation between parasites and Enterobacteriaceae, in 30.4% (7/23) of turtle subjects of this study both parasites and Enterobacteriaceae were detected, whereas in 47.8% (11/23) of turtles exclusively Enterobacteriaceae were detected. In the remaining 21.8% (5/23) of turtles neither parasites nor Enterobacteriaceae were detected (Table 1). No significant relationship was observed between parasites and Enterobacteriaceae (χ^2^ = 2.7951, DF = 1, *p* > 0.05; r_s_ = 0.35321, *p* > 0.05).

## Discussion

The loggerhead sea turtles examined in the present study hosted both Enterobacteriaceae (18/23; 78.3%) and gastrointestinal parasites (11/30; 36.7%).

Concerning Enterobacteriaceae isolation, previous studies conducted on loggerhead sea turtles in Italy have shown variable results in terms of prevalence, probably due to geographical distribution, feeding habits, and health status of the animals, which are all factors known to influence the gut composition [1, 3, 7, 22, 25, 26]. Nevertheless, in line with our results, *Citrobacter* and *Morganella* have been reported as the most abundant Enterobacteriaceae genera, detected by both cultural dependent and independent methods [4, 7, 26].

Despite their ubiquity in the gastrointestinal tract, Enterobacteriaceae have not been constantly detected in the totality of cloacal or faecal samples examined by previous studies [2, 51, 56, 69]. Even when molecular methods were applied, Enterobacteriaceae were not found to be predominant in bacterial communities [4, 7]. However, culture-based methods might favour the outcompetition among microorganisms, possibly explaining the absence of Enterobacteriaceae in five out of 23 samples in the present study.

The role played by bacteria within the sea turtle gastrointestinal tract is still unclear [3]. On one hand, gut bacteria might contribute to digestion and assimilation, influence modulation of the immune system, prevent the overgrowth of noxious species or promote colonization of beneficial species [3, 4]. On the other hand, gut communities might harbour opportunistic pathogens, taking advantage of immune-compromised animals. Indeed, several bacteria have been isolated in association with gastrointestinal lesions, although frequently initiated by other causes (e.g. ingestion of fishing gears, altered gut motility, parasitism) [3, 22, 23, 33, 39, 60]. In particular, the high prevalence of Proteobacteria, to which Enterobacteriaceae belong, has been suggested as a signature of dysbiosis and deteriorated health status in sea turtles and other animals [4, 8].

With respect to the parasitological analyses, the prevalence reported in this study is lower compared to other studies conducted on loggerhead sea turtles in the Mediterranean [28, 40]. In particular, the absence of nematodes was unexpected, as they were detected with prevalence from 16.6 to 71.4% in loggerhead sea turtles from the Tyrrhenian and Adriatic Sea [34, 47, 57]. This finding might be ascribed to the technique, yet it is unlikely, as it has already been successfully used to detect nematode eggs in other reptiles [20, 21, 46]. It is possible that variations in egg shedding rates or the limited availability of intermediate hosts due to seasonal differences of habitat trophic conditions could explain the absence of nematodes in our samples [47, 64]. Another explanation could be sought in different feeding habits of the loggerhead sea turtles, as the turtles in this study were mostly juveniles, whereas the studies of Santoro et al. [47, 53], conducted in the same area, refers to larger animals, probably consuming a wider variety and a greater amount of prey [30, 47, 53, 64].

There is only one report of cestodes in Mediterranean loggerhead sea turtles [58], suggesting their minor role as parasites of loggerhead sea turtles in this area. On the contrary, cestode larvae and cysts were detected in sea turtles from the Atlantic Ocean [29, 32, 64]. However, the technique presented in this study is not suitable for the detection of cestodes, because loggerhead sea turtles serve as intermediate hosts for these parasites, and do not shed their eggs in the faeces.

Similarly, despite the recent evidence of spirorchiid trematodes in loggerhead sea turtles from the Mediterranean, these parasites do not appear to be widespread in this area [36, 48] as they are in the Atlantic Ocean [59, 66]. Indeed, other parasitological studies in the Mediterranean, consistent with our negative results, have not reported the presence of these parasites [5, 34, 47, 58]. Furthermore, detection of spirorchiids, in addition to the faecal examination, requires a thorough examination of the vascular system and almost all tissues, achievable only during necropsy [36, 48].

Regarding other trematodes, all the species detected in this study usually reside in the stomach and upper intestine of different sea turtles species [29, 61]. In loggerhead sea turtles, *Enodiotrema* spp*.* is reported as the most common and widely distributed trematode [29, 64]. The remaining species were more restricted in geographical distribution. It is worth to highlight the detection of *Angiodyctium* spp*.*, because there are no other reports in loggerhead sea turtles, but it has been described in hawksbill (*Eretmochelys imbricata*) and green (*Chelonia mydas*) sea turtles [29, 50, 65].

Concerning coccidia parasites, *Eimeria carettae* is the only species described in loggerhead sea turtles, but there have been no reports since its description [29, 63]. Since coccidia have been considered responsible for mortality events in green sea turtles [15, 27], the present study could raise the interest in this parasite species and its potential pathogenic role in loggerhead sea turtles.

A comparison of the parasitic burden across studies on sea turtles is likely premature, because of the limited data for some species or areas, and the different methods to assess it [35, 36, 61]. Nevertheless, the parasitic burden has generally shown a wide variation, which has been attributed to the influence of several factors, such as life cycle, availability of intermediate hosts, interactions among different parasite species, host immune response and turtle population density [24, 29, 36, 59, 61, 64].

The potential pathological impact of gastrointestinal parasites on sea turtles includes physical injury, inflammatory reaction, resource depletion, and susceptibility to secondary infection, especially in already debilitated hosts [29, 39, 43, 53, 61]. Nevertheless, the parasites that have long co-evolved with their turtle hosts, usually characterized by low pathogenicity, might rather favour the balanced functioning of the immune system, and even be protective against the colonization by less adapted species with high pathogenicity [29, 61].

Given the small number of adults in our sample, and given that the shift between habitats is no longer regarded as a clean change, but rather a transitional period often maintained during adulthood [9, 62], this investigation did not address the association between turtle size and parasitic communities. However, there is to consider that the differences in the parasite communities of sea turtles are mainly ascribed to ecological and ontogenetic factors (e.g. trophic conditions, deep/shallow waters, pelagic/benthic diet, food intake rate) [30, 47].

The lack of correlation between parasites and Enterobacteriaceae, shown in our results, currently joins the already contradicting literature existing on animals and humans [16, 44, 45, 68]. The type of collected sample (cloacal swab), often used in live animals, is not appropriately representative of the bacterial community of the stomach and upper intestine, where the parasites detected in this study usually reside and might have a stronger influence on the local microbiota. This consideration, along with the bacterial isolation through culture-dependent methods, might have influenced the results of Enterobacteriaceae-parasites correlation, limiting this element of the study.

Parasite identification to the species level usually requires thorough morphological examination of adult parasites, mostly available during necropsies through careful organ inspections [28, 36, 47, 64]. Since the present study was conducted on live animals, parasitic identification relied only on examination of eggs by copromicroscopy that did not allow achieving a definitive identification at the species level.

Thorough sensitivity analyses of diagnostic techniques for parasites generally require post-mortem examination [13, 35], and therefore an assessment of the sensitivity of the FLOTAC technique was not conducted in this study which used live subjects. However, the FLOTAC technique provided successful results in reptiles, and in this work was applied for the first time on live sea turtles. The FLOTAC technique is not suited to detect parasites that use sea turtles as intermediate or paratenic hosts, but it is appropriate for an early diagnosis upon admission and for the management (e.g. monitoring quarantine, treatment regime, etc.) of the majority of sea turtle parasitoses. More importantly, given that the egg count should not be considered indicative of the severity of the disease, which might vary depending on the parasite species, the parasitic burden, and the host’s immune system [13, 29, 35, 67], this technique, being applicable to live animals, could relate the clinical manifestation of the parasitosis to a specific agent, improving the knowledge on sea turtle parasitosis, promoting future epidemiological studies, and also allowing proper treatment and management of animals in rehabilitation facilities. Specifically, we suggest performing parasitological analyses on all recovered sea turtles upon admission. Quarantine measures should be adopted whenever there is the possibility for the parasite to complete its life cycle (e.g. open systems, improper filtration) and disseminate infecting parasitic elements among other sea turtles in rehabilitation. A specific treatment regime should be established, once the turtle has been stabilized and might better tolerate medications, on the basis of the parasitological results, in order to ameliorate the health status and welfare of an already stressed animal, and to optimize and reduce the rehabilitation period.

## Conclusions

Free-living sea turtles naturally host bacteria and parasites in their gastrointestinal tract [5, 7, 13, 28, 47, 50, 52, 64]. The present study provided baseline data on Enterobacteriaceae and parasitic composition in live loggerhead sea turtles from the Mediterranean, evidencing *Citrobacter* and *Morganella* as the most common bacterial genera and digenetic trematodes as the most common parasites in these animals. Although bacterial and parasitic communities might have a relatively benign relationship with their host, they might express their pathogenic potential in debilitated animals, such as those in rehabilitation facilities [3, 4, 14, 22, 23, 25, 50, 51, 53, 59]. Therefore, it is advisable to include in the standard work-up of rescued sea turtles a screening procedure for such opportunistic agents, to better evaluate the animal health status in relation to their presence, and to intervene in a timely manner with appropriate treatment, thus improving rehabilitation.

Furthermore, data collected from free-living sea turtles represent a starting point for investigating wild populations. However, further studies are needed to clarify the differences between sea turtle’s normal gut microbiome and pathobiome, especially with the aid of molecular methods [4]. This knowledge would help to restore the host’s gut in rehabilitating sea turtles prior to reintroduction in their natural habitat.

## Methods

### Sampling

A total of 30 loggerhead sea turtles, temporarily kept at the Marine Turtle Research Centre (Stazione Zoologica Anton Dohrn, Portici, Italy), were examined. All the recovered sea turtles were found in near shore environments along the coasts of Italy. Specifically, 20 turtles came from the southwestern coast (area W; from 42°5′18.94″N 11°47′12.62″E to 39°59′55.79″N 15°25′37.37″E), corresponding to the Lazio and Campania regions, whereas ten turtles came from the southeastern coast (area E; from 41°55′43.5″N 15°08′18.8″E to 39°47′37.1″N 18°22′08.1″E), corresponding to the Apulia region (Fig. 1).

Upon admission at the Marine Turtle Research Centre, sea turtles were examined and morphometric parameters were collected. Body Condition index resulted higher than 1.2 for all subjects, indicating a good nutritional status [37]. In accordance with Casale et al., [11], sea turtles were classified, on the basis of their curved carapace length (ranging from 9.2 to 85.0 cm, with average 50.2 cm), as juvenile individuals, with the exception of five adults. In order to perform bacteriological and parasitological analyses, sea turtles were kept in individual tanks, and one cloacal swab sample and one faecal sample were collected for each animal. Cloacal swab samples were collected upon the turtles’ arrival at the Centre, inoculated in phosphate buffered saline (Oxoid) and transported at 4 °C to the laboratory of the Department of Veterinary Medicine and Animal Productions (University Federico II of Naples, Italy). Seven cloacal swabs had to be excluded from this study, because of a suspect of contamination during bacteriological analyses. Faecal samples consisted of the first faeces emitted by the turtles, which were collected in sterile containers and transported, at 4 °C, to the Regional Centre for Monitoring Parasitosis of the Department of Veterinary Medicine and Animal Productions (University Federico II). All samples were preserved at 4 °C, until further analyses, within 24 h. Animal procedures are included in the standard clinical examination and diagnostic investigations of rescued sea turtles, and all animals were rehabilitated and reintroduced in nature, in accordance with the authorization by the Ministry of Environment and Protection of Land and Sea (Protocol n.0042848/PNM 09/08/2013 and Protocol n.0024471/PNM 22/11/2016).

### Bacteriological analyses

Samples in phosphate buffered saline were analyzed in order to isolate and identify members of the Enterobacteriaceae family, following laboratory protocols based on ISO procedures, including the use of control organisms for quality check. Specifically, each sample was transferred into buffered peptone water (Oxoid) and incubated at 25 °C and 37 °C for 24 h. Subsequently, the samples were streaked onto MacConkey agar n. 3 (Oxoid) plates and incubated at 25 °C and 37 °C for 24 h. All isolated strains were primarily identified on the basis of their colonial morphology, lactose metabolism, pigment production, and standard biochemical tests. The isolates were then confirmed using the Analytical Profile Index system (bioMérieux), and the identification to the species level was considered successful when reading provided at least “Very Good id.” (%id > 99.0 and T > 0.5) [38].

### Parasitological analyses

Due to the paucity of faecal material and its dispersion in the individual tank, faecal samples were analyzed using the FLOTAC Pellet Technique [18]. This technique has been successfully used in reptiles for samples with an unknown weight of faecal material [20, 21, 46]. The protocol was applied as previously described [20, 46]. Briefly, each sample was homogenized, filtered (mesh size of 250 μm) and centrifuged in two conical tubes for 3 min at 1500 rpm. Subsequently, the obtained sediments (pellets) were weighted and re-suspended with different flotation solutions (FS): one with FS2 (Sodium Chloride Solution; 1200 s.g.) and the other with FS7 (Zinc Sulphate Solution; 1350 s.g.). The suspensions were used to fill the two flotation chambers of the FLOTAC apparatus, and centrifuged for 5 min at 1000 rpm. Then, the apical portions of the flotation chambers were horizontally rotated and analyzed under a light microscope at 10X and 40X magnifications (Leica DFC 490), in order to count, photograph, measure, and evaluate the parasitic elements (eggs and oocysts), in accordance with the current literature [29, 61]. For each animal, the results were expressed as eggs/oocysts per gram of faeces (EPG/OPG), calculated using the following formula: EPG/OPG = (N × 1.2)/wp where N is the number of eggs/oocysts counted and wp is the weight of the pellet [20, 46].

### Statistical analyses

The possible association between parasites and Enterobacteriaceae was evaluated. Chi square analysis and Fisher Exact test were performed to evaluate the relationship between Enterobacteriaceae positivity and parasites positivity, whereas Spearman’s r_s_ correlation was performed to evaluate the relationship between Enterobacteriaceae detection and parasitic burden (EPG/OPG). Statistical analyses were performed with Past (Hammer and Harper) and statistical significance was set at *p* < 0.05.

## Data Availability

The datasets used and/or analysed during the current study are available from the corresponding author on reasonable request.

## Abbreviations

CI: Confidence interval
DF: Degrees of freedom
EPG: Eggs per gram of faeces
FS: Flotation solution
OPG: Oocysts per gram of faeces
SE: Standard error
WP: Weight of pellet
